# Efficacy of contact tracing for the containment of the 2019 novel coronavirus (COVID-19)

**DOI:** 10.1136/jech-2020-214051

**Published:** 2020-10-01

**Authors:** Matt J Keeling, T Deirdre Hollingsworth, Jonathan M Read

**Affiliations:** 1 Zeeman Institute (SBIDER), University of Warwick, Coventry, UK; 2 Big Data Institute, University of Oxford, Oxford, UK; 3 Centre for Health Informatics, Computing and Statistics, Lancaster Medical School, Lancaster University Faculty of Health and Medicine, Lancaster, UK; 4 Institute of Infection and Global Health, University of Liverpool, Liverpool, UK

**Keywords:** communicable diseases, Disease modeling, Epidemiology, public health policy

## Abstract

**Objective:**

Contact tracing is a central public health response to infectious disease outbreaks, especially in the early stages of an outbreak when specific treatments are limited. Importation of novel coronavirus (COVID-19) from China and elsewhere into the UK highlights the need to understand the impact of contact tracing as a control measure.

**Design:**

Detailed survey information on social encounters from over 5800 respondents is coupled to predictive models of contact tracing and control. This is used to investigate the likely efficacy of contact tracing and the distribution of secondary cases that may go untraced.

**Results:**

Taking recent estimates for COVID-19 transmission we predict that under effective contact tracing less than 1 in 6 cases will generate any subsequent untraced infections, although this comes at a high logistical burden with an average of 36 individuals traced per case. Changes to the definition of a close contact can reduce this burden, but with increased risk of untraced cases; we find that tracing using a contact definition requiring more than 4 hours of contact is unlikely to control spread.

**Conclusions:**

The current contact tracing strategy within the UK is likely to identify a sufficient proportion of infected individuals such that subsequent spread could be prevented, although the ultimate success will depend on the rapid detection of cases and isolation of contacts. Given the burden of tracing a large number of contacts to find new cases, there is the potential the system could be overwhelmed if imports of infection occur at a rapid rate.

Contact tracing is the main public health response to importations of rare or emerging infectious diseases, and was implemented in the UK during the ‘containment stage’ of the 2009 influenza pandemic.^1^ In more recent years, contact tracing was also a valuable tool following the importation of the Ebola virus disease into the UK in 2014^2^ and the cases of monkeypox in the UK in 2018.^3^ In general, contact tracing is a highly effective and robust strategy given sufficient resources. The main advantages are that it can identify potentially infected individuals before severe symptoms emerge, and if conducted sufficiently quickly can prevent onward transmission from the secondary cases. Contact tracing has proven hugely successful in the treatment of sexually transmitted infections, where the definition of a contact is relatively straightforward, where the infection is often asymptomatic and where the time-scales of transmission are slow.^4^ In contrast, the use of contact tracing for novel invading pathogens has received less quantitative consideration, in part due to greater uncertainties over social contact structure (although see^5 6^) Modelling studies have often focused on quantifying the importance of pre-symptomatic and pre-tracing infectiousness, but are usually based on statistical distributions of contact networks.^7 8^ Here we leverage detailed social network data from the UK to model both transmission and the act of tracing, and identify the implications of early contact tracing for containment of a novel pathogen, using parameters for the novel coronavirus (COVID-19).^9 10^

## METHODS

We characterised contact patterns in the UK using a postal and online cross-sectional survey, which asked participants to report the number of social encounters with unique individuals during a given day, as well as the duration and typical frequency of those encounters.^11 12^ In total, 5802 respondents reported more than 50 000 encounters—one of the biggest studies of its kind to date. The definition of a contact used in the survey was a face-to-face conversation within 3 m or where skin-on-skin touch occurred. This will naturally include all conversational contacts within 2 m (the standard definition for a contact for COVID-19 tracing), but is unlikely to represent a significant overestimate. The encounter patterns of this study were in good qualitative agreement with other similar studies of social interactions.^13 14^

10.1136/jech-2020-214051.supp1 Supplementary data

**Figure 1:**
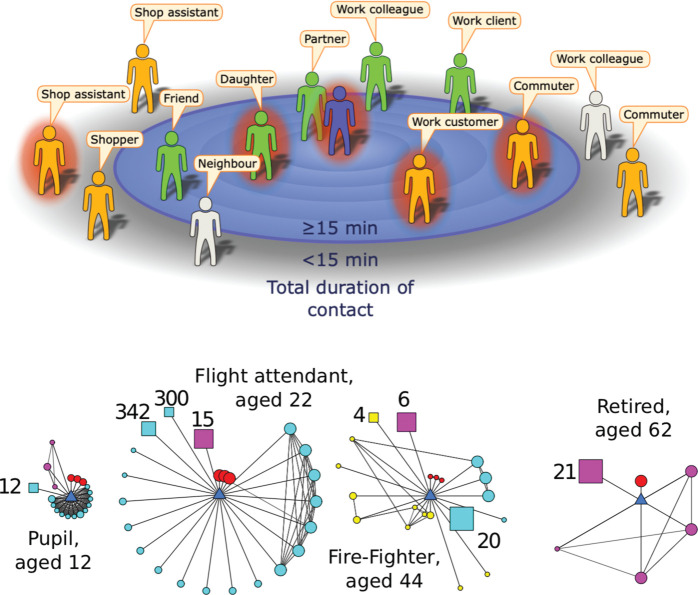
(A) Cartoon example of the encounters made during a day by an infectious index case (central figure) with contacts positioned by their total contact duration. Here, the definition of a contact is someone with whom the index case encountered for 15 min or longer.^15^ Some contacts will be identifiable (green), while others will be unidentifiable (orange). A definition of contact that is too restrictive and inappropriate for the infection means some encounters may fail to meet the definition yet may be at risk of infection; these excluded contacts could be identifiable (light grey) or unidentifiable (orange). (B) Examples of ego-centric networks collected by the survey.^11 12^ The participant (ego) is the blue central triangle; circles represent individual contacts, squares represent groups of contacts (size of group indicated). Colours represent social settings of encounters (red=home, cyan=work/school, yellow=travel, pink=other). Larger symbol sizes represent longer contact durations, while a closer proximity to the ego indicates the contact is more frequently encountered.

In this study, the daily encounter data were first extrapolated to generate a pattern of contacts over a 14-day period (replicating random encounters and increasing the total duration of associated regular contacts), to act as the basis for transmission and contact tracing simulations (see online appendix 1 for more technical details). Using these extrapolated data, we can determine which interactions satisfy a given definition of a close contact for the purpose of contact tracing. From our social encounter survey, we consider all reported contacts of 15 min or more as meeting the close contact definition. From our social encounter data, we can also distinguish interactions with people who could be later identified and traced, from those with unidentifiable strangers (schematic figure 1); although we note that electronic means of tracing should be able to trace these individuals.^15^ We assume that all contact of longer than 1 hour or repeated contacts can be identified and traced, whereas shorter duration encounters with people met for the first time are strangers who are unidentifiable and therefore untraceable.

The second element of the simulation is to determine who gets infected from a source case chosen representatively from the survey respondents. This transmission process is stochastic, accounting for both the time spent with each contact and the infectivity on each day (see online appendix 1). The transmission rate to a contact is scaled to generate the required basic reproductive ratio, *R*_0_. Taken together these two predictions allow us to bound the efficacy of contact tracing.

## RESULTS

**Figure 2:**
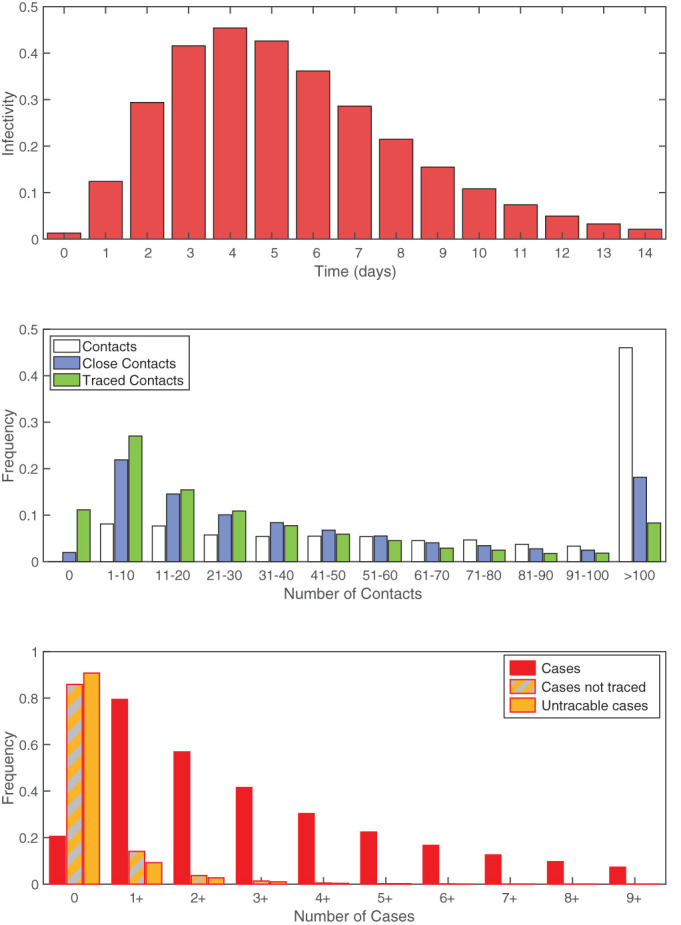
Distributions associated with transmission and contact tracing. (A) Infectivity over time based on an SEIR model with a latent period of 4 days (Erlang distribution with shape 3), infectious period of 2 days, *R*_0_=3.^9^ (B) Frequency distribution of the number contacts over a 14-day period using colours from figure 1A: white is all contacts; blue are those matching the >15 min definition of a close contact; green are those matching the definition that are also assumed to be identifiable (met previously or for more than 1 hour), and therefore traceable. (C) Frequency distribution of the number of secondary cases per index case, again using colours from figure 1A: red is all secondary cases; grey and orange are those that are not traced either through failing to meet the definition of a close contact or because they are assumed to unidentifiable; orange are all secondary cases that are shorter than 15 min or unidentifiable.

One of the most notable features of human social contacts is the huge variability in the number and strength of contacts—which is reflected as variation in both the number of secondary cases and the number of individuals that match the contact tracing definition (figure 2). Using preliminary estimates of COVID-19 transmission (average latent period 4 days, average effective infectious period 2 days, *R*_0_=3, and assuming a simple SEIR formulation^9^) we compute the distribution of epidemiological, social and contact tracing characteristics across the population. Extrapolating the data from the social contact survey suggests that the average number of contacts over a 14-day period is 217, although the distribution is significantly over-dispersed (with a median of 90 and around 3% of individuals having >1000 total contacts). Of these total encounters, an average of 59 contacts (27%) meet the definition of a close contact (in contact for >15 min,^16^) and of these close-contacts we predict an average of 36 (61%) to be individuals who can be identified by the infected case and can therefore be traced. This is comparable to early reports from Singapore^17^ and Taiwan^18^ where 84 and 100 confirmed cases led to 2593 and 2761 contacts being traced, respectively (approximately 31 and 27 contacts per case). Therefore, simply considering social contacts, it is clear that there are very many short duration contacts that do not meet the definition of a close contact, and although unlikely to become infected may pose a risk due to their greater abundance. As expected, tightening the definition of a close contact can dramatically lower the number of contacts that would need to be traced: identifying contacts from 7 days prior to detection reduces the average number of contacts to 128 (median 55).

Given that the risk of infection increases with the duration of contact, the distribution of cases effectively represents a biased sample of all contacts. As expected, given the model assumptions, the expected number of total secondary cases agrees with the assumed *R*_0_ (mean=3, median=2, and 95th percentiles 0–10). Given that secondary cases are most likely to be those contacts of the longest duration, we predict that 95% of cases match the definition of a close contact. However, not all of these contacts will be identifiable; assuming that all repeated contacts and contact of longer than 1 hour can be traced, we predict that 93% of all cases meet the definition and can be identified. However, because of the extreme heterogeneity in contacts between individuals and the stochastic nature of transmission, we would still expect approximately 15% of all primary cases to generate at least one secondary case that is not traced and 10% to generate a secondary case that cannot even be identified. Similarly, we would expect around 3% (10%/*R*_0_) of detected cases to not be able to identify their infecting individual. Neither of these results should be viewed as a failure of contact tracing, merely a reflection of the uncertainties in the approach. Aggregating across all individuals, and under the optimistic assumption that all the contact tracing can be performed rapidly such that all close contacts are traced before they become infectious, we expect such highly effective contact tracing to reduce the basic reproductive ratio *R*_0_ from 3 to 0.18—enabling the outbreak to be contained (figure 2). Less effective tracing (tracing only a random fraction of contacts) would lead to a linear scaling in the reduction of the *R*_0_ such that over 71% of contacts need to be traced to reduce *R*_0_ below 1 and control the outbreak. This efficacy would need to be increased if contacts were not traced and isolated before they were infectious (a problem exasperated by pre-symptomatic transmission), or could be reduced if the higher risk/longer duration contacts were preferentially traced.

**Figure 3:**
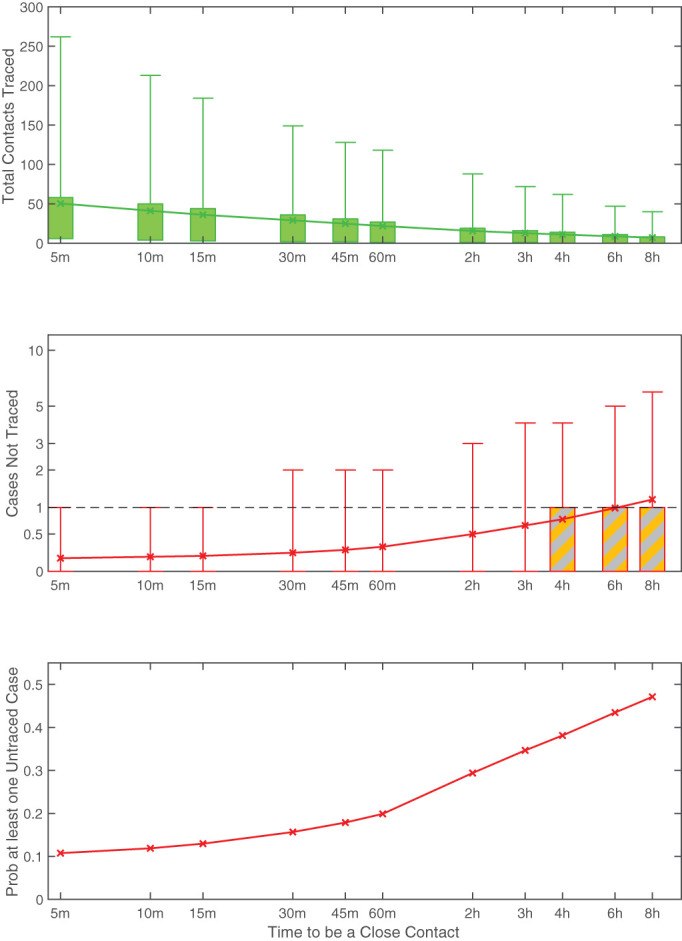
Impact of different assumptions for the definition of a close contact on: (A) the total number of contacts traced per index case; (B) the number of secondary contacts that are not traced per index case; and (C) the probability that at least one secondary case is not traced per index case. For (A) and (B) the crosses mark the mean value, boxes contain the 50th percentiles while bars contact the 95th percentiles, and colours correspond to those in figure 1A—distributions are across all respondents to the survey and across stochastic realisations. (Based on an SEIR model with latent period 4 days, infectious period 2 days, *R*_0_=3^9^).

Rapid and effective contact tracing can therefore be highly effective in the early control of COVID-19, but places substantial demands on the local public health authorities. Each new case requires an average of 36 individuals to be traced, with 8.7% of cases having more than 100 close traceable contacts (figure 2). We therefore consider the implications of changing the definition of a close contact. Clearly, a more strict definition of a close contact (requiring more contact time) reduces the burden on the health services as fewer contacts need to be traced, but also increases the risk of cases being missed. Figure 3 provides a quantitative assessment of changes to the close contact definition. Definitions requiring more than 4 hours of contact are unlikely to control an outbreak as the expected number of untraced second cases is greater than one. This therefore places a strict upper bound on the level of contact tracing required. The added benefit from definitions shorter than 1 hour has relatively little impact on the mean number of untraced cases (figure 3B), but does reduce the probability that some untraced contacts occur.

**Figure 4:**
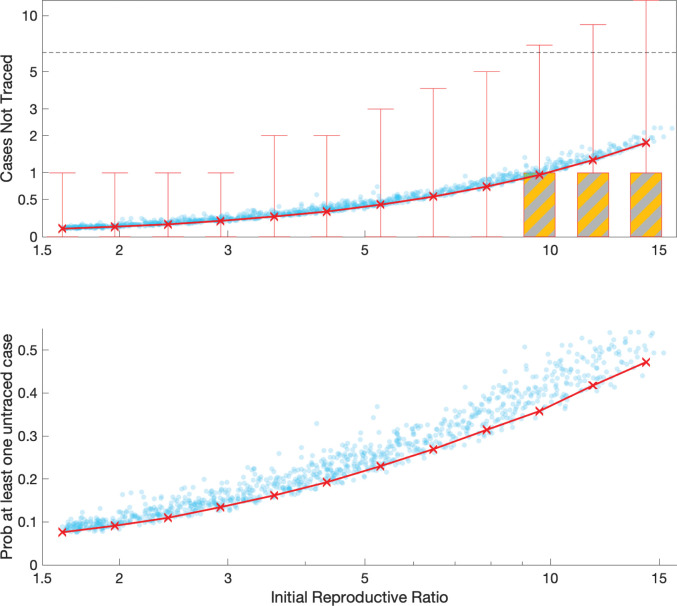
Impact of different values for the initial reproduction number of the primary case; on (A) the number of secondary contacts that are not traced, and (B) the probability that at least one secondary case is not traced. changing this reproduction number does not affect the number of contacts traced. For (A) the crosses mark the mean value, boxes contain the 50th percentiles while bars contact the 95th percentiles—distributions are across all respondents to the survey and across stochastic realisations. (Main results are based on an SEIR model with a latent period of 4 days (and three latent classes), and an infectious period of 2 days; other points, in blue, use a latent period chosen from a lognormal distribution^10^ and an infection period between 2 and 3 days, and are based on a model with one, two or three latent and infectious classes).

Throughout we have used a value of *R*_0_ that represents a population-level average once the local infection has become established. However, the first invasion into any new population or social setting generally has a larger than expected number of secondary cases. The first invader enters a completely susceptible population; moreover all their close contacts (eg, family members) are susceptible. In contrast, due to the clustering of contacts, most secondary cases will be in a landscape with a depleted number of susceptibles—as close contacts such as family members will already have been exposed to the primary case. This susceptible depletion in the local social network may help to explain the change in *R*_t_ over time reported for COVID-19.^18^ We therefore consider the impact of different values of the initial reproductive ratio (figure 4), which could capture this social aspect, or could represent heterogeneity between individuals in the amount of virus shed, or could inform about innate differences in behaviour between China and the UK. Given the strong biasing of transmission towards long-duration contacts, the impact of varying the initial reproductive ratio is less extreme than might be expected; it is only for the highest values of the initial reproductive ratio simulated (>9.8) that contact tracing fails to find more than one case such that infection can escape. We also consider sensitivity to alternative formulations and parameter values for the epidemiological dynamics, and conclude that the success of contact tracing against COVID-19 is predominantly driven by the initial reproduction ratio.

## CONCLUSIONS

Mathematical models have an important role to play in preparedness for novel infectious diseases, allowing policymakers to plan for potential public health scenarios before they arise. However, in such scenarios reliable data are often limited, so predictions of long-term dynamics are generally associated with wide CIs. In contrast, while short term predictions are subject to greater stochasticity, the distribution of possible behaviours can be readily captured. Here we have investigated contact tracing of a close-contact pathogen, using the 2019 novel coronavirus (COVID-19) as the example, and considered the efficacy of contact tracing as a control measure. This work brings together a detailed survey of social encounters together with bespoke mathematical modelling of the transmission and tracing processes. Given the substantial heterogeneities present in social encounters (both in terms of duration and number), mathematical models are vital to interpret the interplay between a low number of high–risk encounters (eg, household members) and the high number of low–risk less–identifiable encounters (eg, commuters or retail customers).

Throughout this work we have used a simple definition of a close contact as anyone being within 3 m of an infected individual for 15 min or more, over a 2-week period—relating to the stipulation in our earlier study. This is likely to be a slight overestimate compared to the UK definition which uses a 2 m distance rule.^16^ However, other countries and regions have subtly different protocols^19 20^ with critical distances ranging from 1.5 to 2 m and times from 10 to 15 min. Our assumption of tracing all contacts in a 2-week period is likely to be pessimistic, with most countries now adopting an interval from 2 days before symptoms to isolation of the patient. Under our default definition, there are unlikely to be many unidentified secondary cases, although the burden of tracing all contacts could be large. Relaxing the definition of a contact (such that longer contact durations are needed) lessens this burden, but at the greater risk of undetected cases (figure 3). Surprisingly, moderate changes to the reproductive ratio, within the bounds estimated from early data,^9 10 21 22^ or changes to the time course of infectivity are predicted to have a relatively modest impact on the success of contact tracing, illustrating the robustness of this control measure (figure 4).

Our model has addressed the simple and optimistic question of whether rapid and complete contact tracing is sufficient to identify secondary infections. The public health reality of contact tracing is more complex, and depends on the relative timing of events and the management of identified contacts. For contact tracing to be an effective public health measure requires most secondary cases to be discovered and isolated before they become infectious; hence the time from the primary case becoming infectious to the tracing of their contacts needs to be shorter than the incubation period. Longer time scales would allow tertiary cases to be infected and potentially increase the scale of tracing required. In addition, those contacts that are traced either need to be effectively screened for infection and quarantined or otherwise isolated so that they do not pose a risk to others.

We have also assumed that all index infections are identified as cases and start the process of contact tracing, leading to the tracing of all identified contacts. This is clearly an extremely optimistic assumption: not all infections are symptomatic so may go undetected, and not all those who are symptomatic will seek medical help; and not all identified contacts can be traced sufficiently rapidly to prevent further spread. Therefore, while contact tracing has the potential to contain COVID-19 (and other close-contact pathogens) during the early stages of invasion the ultimate success relies on the speed and efficacy with which suspect contacts can be contained and the capacity for contact tracing.

Contact tracing can also be used later in an outbreak to assist with other control methods in reducing the number of cases.^23^ In this scenario, other factors become important: the type and number of contacts are likely to be extremely different for countries in or exiting lockdown; the effective reproductive ratio is likely to be far lower; and household contacts may already self-isolate making tracing irrelevant. These considerations mean that contact tracing needs to be less effective to control the infection (more readily bringing the reproductive ratio below 1) but is likely to have diminished impact due to the existence of other measures.

**What is already known on this topic**

- Contact tracing is known to be highly effective for diseases that spread slowly by close contact, and hence is used for many sexually transmitted infections.
- Quantitative predictions of contact tracing have generally focused on the speed of tracing, and used assumed contact patterns. These studies have shown that control is most effective when the latent period is long and the disease transmits slowly.

**What this study adds**

- By considering the distribution of close contact encounters, we are able to predict the efficiency of contact tracing in identifying secondary cases.
- The UK definition of a close contact (15 min or more, within 2 m) is sufficient to contain imports of infection but at the cost of tracing many uninfected contacts.
- We would expect 10–15% of cases to generate at least one unidentified secondary case which would need detecting by other means.
