# Supplementary material for: Efficacy of contact tracing for the containment of the 2019 novel coronavirus (COVID-19)
Source: J Epidemiol Community Health. 2020 Oct 1;74(10):861–6. doi: 10.1136/jech-2020-214051 (PMC7307459; doi:10.1136/jech-2020-214051)
Supplement: Supplementary data [file jech-2020-214051s001.pdf]

**APPENDIX 1**

The social contact survey conducted in 2009-10 recorded the following information on all contacts (or group of contacts) on a given day:
**Location** of contact: Home; Work/School; Travelling; Other
**Duration** of contact on this day: <10mins, 10-30 mins, 30-60 mins, >60 mins
**Frequency** of meeting this contact: 4 or more days a week, 2-3 days a week, once a week, less often, first time.
**Size of Group**, for contacts that are grouped together, such as total number of customers served in shop.
As such this provides a very detailed picture of the ego-centric contact network in the UK, capturing the substantial heterogeneity – the mode number of contacts is 5 per day, with a mean of 26.75 but some individuals report more than 1000.

This survey is analysed in Danon et al [11,12] where the structure of the network and the epidemiological consequences are studied in more detail. Here we use the methodology outlined in Danon et al 2012 supplementary material [11] to impute the likely network across time scales of longer than one day:
**Step 1**. Groups of contacts are expanded to be contacts with multiple individuals with the same defining characteristics.
**Step 2**. The duration of contact is well approximated by a stretched exponential distribution $\exp(-A_L t^{B_L})$ for contact time *t*, where the parameters $A_L$ and $B_L$ depend on the location *L*. Therefore for each contact (or individual in a group of contacts) *c,* we can chose a contact time $T_{c,d}$ for each day *d*, sampling from the appropriate distribution conditional on the true recorded duration.
**Step 3**. Accounting for frequency of contact, determine if the contact is repeated (for the same contact) or replicated (generating a new contact). For each reported contact we determine how often they are encountered during a *D*-day period, using the frequency reported. For frequencies of 4-7 days a week or 2-3 days a week, the assumed number of days with this contact is sampled randomly from appropriate interval. Except when a contact is assumed to encountered 7 days each week, contacts are replicated. Hence contacts that are met for the first time or less than once a week, are replicated for each day and new contact times chosen. For contacts that are met more often, replicate contacts are generated for all days on which the reported contact is not present – so contacts that are met 2-3 times a week will be replicated 3 or 4 times. For days on which a particular contact is not present the associated contact time $(T_{c,d})$ is set to zero.

This therefore generates a larger set of contacts *c* (for each respondent, *r*) with an associated contact time on each day $T^r_{c,d}$. We can use this information to define the key measurables of local transmission and contact tracing.

We define a close contact of *r* as all contacts *c*:

$$C_r = \left\{ c \,\middle|\, \sum_{d \in D} T^r_{c,d} > T \right\}$$

In the UK, we have defined the total contact time *T* as 15 minutes over a duration *D* of two weeks before detection and isolation of individual *r* [15]. This refers to all those individuals within the blue circle in Figure 1a. However, not all of these will be identifiable (for example commuters on public transport or people served in a shop), we therefore define the non-identifiable contacts of respondent *r* as:

$$N_r = \left\{ c \,\middle|\, \text{Frequency}_c = \text{first time } \cap T^r_{c,d} < 1 \text{ hour} \right\}$$

So this is all those contacts that were met for the first time and for less than one hour (coloured orange in Figure 1a). Hence the set of all contacts that are both identifiable and meet the close-contact definition are:

$$Traced_r = C_r \setminus N_r$$

The probability of transmission to contact *c* from respondent *r* is then calculated as:

$$\text{Prob(infection } r \rightarrow c) = 1 - \exp\left(-\sum_{d \in D} \tau_d \, T^r_{c,d}\right)$$

Where $\tau_d$ is an estimate of the transmission rate from individual *r* on day *d*.

Hence the expected number of secondary cases from individual *r* is:

$$R^r = \sum_{c} \left[1 - \exp\left(-\sum_{d \in D} \tau_d \, T^r_{c,d}\right)\right]$$

And the expected number of cases that are not traced as:

$$\hat{R}^r = \sum_{c \notin Traced_r} \left[1 - \exp\left(-\sum_{d \in D} \tau_d \, T^r_{c,d}\right)\right]$$

In this work, we have considered the uncertainty in the extrapolation of the network, the uncertainty in the stochastic transmission process and the between individual variability.
